# 

**Published:** 1836

**Authors:** 


### CONTENTS OF VOL. X.

### NO. XXXVII.

### ESSAYS AND CASES

Art. I.—Cæsarean Operation. By R. Estep, M. D. p. 13

II.—Acetate of Lead, in Cholera and Dysentery. By D. A. B. Brice, 19

III.—Rheumatico-Catarrhal Epidemic. By H. Perrine, 26

### REVIEWS AND BIBLIOGRAPHICAL NOTICES.

IV.—Medical Jurisprudence, 34

V.—Sources of Health and Disease in Communities, 86

### MISCELLANEOUS INTELLIGENCE.

#### ANALECTIC.

A succinct account of the Hospitals, Medical Institutions, etc., of Paris, 97.—Of the refrigerant method in the treatment of Nymphomania, 105.—New Therapeutic considerations concerning the Employment of the Oil Croton Tiglium, ib.—Rules to be followed connected with premature delivery of the cord, 106.—Of the treatment of Fractures of the lower extremities without confining the patient to bed, 107.—Method of forming an artificial pupil, without injury to the Crystalline Lens or its capsule, ib.—Amourisis from Metalic Cholic, 111.—Romnade for the cure of enlarged Tonsils, 112.—Hemoptesis cured by Creosote, ib.—Hydrocele of the neck, with cases and observations, ib.—Radical cure of Varicocele, 117.—Geitre Extirpation, cure, 117. On the removal of Sequestra without an operation, 118.—On the effects of poison on the animal system, 119—Case of poisoning by Arsenic, 121.—On the climate of Madeira and its true value in consumption, 131.—Medical instruction in Paris, 132.—Case illustrative of the effects of Iodine used externally, 133.—Bleeding in the cold stage of Intermittent Fever, 135.—On the Stimulating effects of cold water, 137.—Various effects of Iodine, 139. Account of the Burmese practice of Midwifery, 140.—Experiments upon the means of producing an artificial Cataract, 144.—New process for the operation, Artificial pupil, ib.—A case of Introsuspection in which an operation was successfully resorted to, 145. On Delirium Tremens, 146.—Dr. Graves on the Chloride of Sodium in Fever, 146.—Tyhpus in the young, 147.

#### ANALYTICAL.

Art. 1.—Vaccination, variola, varioloid, 149.—II. Creosote, its preparation physiological action, medical employment and mode of administration, 152.—III. Syphilitic sore of Urethra, 159.—IV. Hospitals and their regulations, 160 V. Tartar Emetic in Obstetric Practice, 161.

#### ORIGINAL.

Art. I. Exchange Journal, 165.—II. Our periodicals, 166.—III. Professor Parker, 156.—IV. Summer Lectures, 167.—V. Health of Cincinnati, 167. VI. Cincinnati Medical Society, 168.

### NO. XXXVIII.

### ESSAYS AND CASES.

ART. I.—Cases from the Diary of a Physician, p. 169
II.—Researches on Phthisis, by M. Louis, 173
III.—Cases of Pectoral Disease, by O. M. Herron, M. D. 201

### REVIEWS AND BIBLIOGRAPHICAL NOTICES.

IV.—Principles of Pathology and Practice of Physic, 208
V.—Researches in Medicine and Medical Jurisprudence, 245
VI.—General Therapeutics, 251

### MISCELLANEOUS INTELLIGENCE.

ANALECTIC.

1. Puerperal Convulsions, p. 253.—2. Corinoma of the Tongue, 255.—3. Sketch of the case of Dr. Crosby. 255.—4. Cold baths in Cholera, 257.—5. Examination of the head of Fieschi, the French assassin, 257.—6. Double vision, 258.—7. Modification of System by age, 258.—8. Effects of Nux Vomica and Aconite on Animals, 261.—9. Pathology of Rheumatism, 262.—10. Alumina in Cholera, and Sulph. Cupri in Softening of Stomach, 263.—11. Clinical Observations on the effects of certain Medicines on the heart, 266.—12. Alum in Typhoid Fevers, 267.—13. Turpentine in Sciatica, 267.—14. Purulent Metastasis, 267.—15. Temperature of Sinapisms, 268.—16. Poisoning by Arsenic, cured, 268.—17. Antidote for Arsenic, 269.—18. Fumigations in Hooping Cough, 270.—19. Treatment of Scald Head, 271.—20. Treatment of luxations of Clavicle, 273—21. Medical Jurisprudence and Toxicology, 273—22. Ulcer of the Pylorus, 274.—23. Singular dose of Lead in Tibra, 277.—24. Elephantiasis of the Arabians, 278.—25. Remarkable Monstrosity, 280.—26. Use and Abuse of cold application, 280.—27. Recovery from Asphyxia in an Infant, 282.—28. Sleeplessness and its Treatment, 283.

ANALYTICAL.

1. Consultation with Quacks, 293.—2. Quackery, 296.—3. Medical Students, 297.—4. British and Foreign Review, 300.—5. Creosote, 305.—6. Fungus Hamatodes, 307.—7. Operation for Inguinal Aneurism, 308.

ORIGINAL.

Traveling Memoranda from the Editor, 311.

### NO. XXXIX.

### ESSAYS AND CASES.

ART. I.—Epidemic Erysipelas, p. 325
II.—Fracture of Cervical Vertebra, 328
III.—Secretion of Milk suspended by Sassafras Tea, 332
IV.—Diseases of the Antrum Maxillare, 333
V.—Great Ascitic Effusion, 343
VI.—Bleeding in the Chill of Intermittents, 345

### REVIEWS AND BIBLIOGRAPHICAL NOTICES.

Art. VII.—Comparative Physiology, 350
VIII.—Importance of Physical Signs, 369
IX.—Cyclopædia of Practical Medicine, 385
X.—American Cyclopædia of Practical Medicine, 414

### MISCELLANEOUS INTELLIGENCE.

ANALECTIC.

1. On Ligature of Arteries in case of Aneurism, p. 421.—2. On the Arteries of the Conjunctiva, 424.—3. On the power by which the head of the thigh bone is retained in its Socket 425.—4 Asthma, 426.—5. New mode of administering Balsam Copaiba, 431.—6. On the Effects of Indigo in Epilepsy, and other Spasmodic Affections, 432.—7. Relation between the state of the Pulse, Respiration and Temperature of the body, in diseases, 434.—8. Report on Vaccination in France during 1833, 437.—9. On the Treatment of Inflammation of the Testicles by means of Compression, 440.—10. Suicide in the Canton of Geneva, 444.—11. Detection of Sugar in the Blood in Diabetes, and on the best mode of separating the Substances from the Urine, 446.—12. Experiments on the action of Oxalic Acid, 447.—13. On the present state of Medicine in Denmark, 449.—14. Case of poisoning by Arsenic, treated with the Hydrated Peroxide of Iron, 466.—5. Medical properties of Creosote, 466.

ORIGINAL.

1. Traveling Memoranda from the Editor, 469. 2. Professional Observations for the year 1837, 476.—3. Convulsions of Children, 477.—4. Scalding by Steam, 478.—5. Cincinnati Eye Infirmary, 479.—6. Summer Medical Instruction, 480. —7. Appointment.

---

### NO. XL.

---

### ESSAYS AND CASES.

Art. I.—Wounded Intestines, p. 481
II.—Diseases of Antrum Maxillare, 493
III.—Extracts from the Diary of an Old Physician, p 500
IV.—Acetate of Morphia in Burns, 505
V.—Borate of Sodae in Dysmenorrhœa, 508
VI.—Spinal Irritation, 513
VII.—Cold Dash in Congestive Intermittents, 519

### REVIEWS AND BIBLIOGRAPHICAL NOTICES.

VIII.—Phthisis Pulmonalis, 528
IX.—Cerebro Spinal Nerves, 562
X.—Elementary Forms of Disease, 564

### MISCELLANEOUS INTELLIGENCE.

ANALECTIC.

1. Use of Belladona in retention of Urine, Spasmodic Contraction of the Uterus, etc. 565, 566.—3. Two cases of Strangulated Hernia, cured by Belladonna Ointments, 567.—4. Influence of atmospheric heat in cure of Wounds, 568.—5. Chlorine Gass for the cure of Hydrocele, 571.—6. Traumatic Opthalmia, 572. —7. Cæsarean Operation performed three times on same Subject, 574.—8. Connection between Hypertrophy of Heart and Apoplexy, 578.—9. Frontal Neuralgia, thirty cases of, ib.—10, Paralysis cured by Electricity and Galvanism, 580.—11. Magnetism in Gout, 581.—12. Proportion of Labors requiring Instruments, 582.—13. Absorption of Placenta, 583.—14. Prolapsus Uteri, 583.—15. Equilibrium of Population and Sustenance Demonstrated, 558.—16. Hyd. Perox. Ferri., antidote against Arsenic, 585.—17. Experiments on Brain, Spinal marrow and nerves, 587.—18. Arteries leading to Inflamed parts, 591.—20. Observations and experiments on functions of Cœcum, 594.—21. Chronic Laryngitis, 596.—22. Oil Croton applied in Chronic Laryngitis, 597.—23. Diseases Simulating inflammatory attacks, 598.—24. Atmosphere in Relation to Madeira, 602.—25. Physiology of Vomiting, 605.—26. Incontinence of Urine, 612.

ANALYTICAL.

I. Doctors, Quack Doctors, and Doctor Quacks, 614.

ORIGINAL.

Medical Department of Cincinnati College, 623.—Spina Bifida cured by Ligature, 631.—Case of Congenital artificial Joint in Ossa Femores, 633.—Hints on venesection in Chill of Intermittents, 634.—New Method of tying divided arteries, ib. Extraordinary number of Convulsions in a Child, 636.

